# Rapid and Cost-Effective Quantification of Glucosinolates and Total Phenolic Content in Rocket Leaves by Visible/Near-Infrared Spectroscopy

**DOI:** 10.3390/molecules22050851

**Published:** 2017-05-20

**Authors:** Eva María Toledo-Martín, Rafael Font, Sara Obregón-Cano, Antonio De Haro-Bailón, Myriam Villatoro-Pulido, Mercedes Del Río-Celestino

**Affiliations:** 1 Department of Genomics and Biotecnology, IFAPA Center La Mojonera, Camino San Nicolás, La Mojonera 1, 04745 Almería, Spain; ortiztoledo@hotmail.com; 2 Department of Food and Health, IFAPA Center La Mojonera, Camino San Nicolás, La Mojonera 1, 04745 Almería, Spain; rafaelm.font@juntadeandalucia.es; 3 Department of Agronomy and Plant Breeding, Institute of Sustainable Agriculture, (CSIC), Alameda del Obispo s/n, 14080 Córdoba, Spain; saraobregon@ias.csic.es (S.O.-C ); adeharobailon@ias.csic.es (A.D.H.-B); mvillatoro@ias.csic.es (M.V.-P.)

**Keywords:** glucosinolates, rocket, *Eruca vesicaria*, visible spectroscopy, near infrared, spectroscopy, chemometrics

## Abstract

The potential of visible-near infrared spectroscopy to predict glucosinolates and total phenolic content in rocket (*Eruca vesicaria*) leaves has been evaluated. Accessions of the *E. vesicaria* species were scanned by NIRS as ground leaf, and their reference values regressed against different spectral transformations by modified partial least squares (MPLS) regression. The coefficients of determination in the external validation (R^2^VAL) for the different quality components analyzed in rocket ranged from 0.59 to 0.84, which characterize those equations as having from good to excellent quantitative information. These results show that the total glucosinolates, glucosativin and glucoerucin equations obtained, can be used to identify those samples with low and high contents. The glucoraphanin equation obtained can be used for rough predictions of samples and in case of total phenolic content, the equation showed good correlation. The standard deviation (SD) to standard error of prediction ratio (RPD) and SD to range (RER) were variable for the different quality compounds and showed values that were characteristic of equations suitable for screening purposes or to perform accurate analyses. From the study of the MPLS loadings of the first three terms of the different equations, it can be concluded that some major cell components such as protein and cellulose, highly participated in modelling the equations for glucosinolates.

## 1. Introduction

Rocket is a herb originated in the Mediterranean region [1], where the aerial part is mostly consumed fresh as salad, but widely distributed all over the world [2]. The term “rocket” refers mainly to *Eruca* and *Diplotaxis* genera within the *Cruciferae* family. Throughout more than 20 centuries, traditional medicine has attributed to rocket plants a number of health-promoting or therapeutic properties, such as antiphlogistic, depurative, diuretic, digestive, aphrodisiac and rubefacient [3]. 

*Eruca* contains a range of health-promoting phytochemicals including carotenoids, vitamin C, fibres, polyphenols, and glucosinolates (GLs) [4]. GSLs are evolutionarily recent secondary metabolic products having arisen million years ago [5], acting as stimulants or deterrents to insects and herbivores [6]. They are known in only a few angiosperm families of the order *Brassicales*, which includes the *Brassicaceae* family, and of which *Eruca* is a member [7]. 

**Figure 1:**
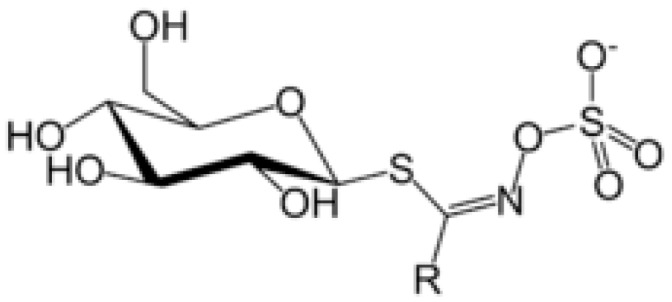
General structure of glucosinolates. R denotes the variable side chain derived from amino acids.

Glucosinolates have a well-defined structure with a side-chain (R-group) and D-glucopyranose as a β-thioglucoside attached to carbon atom no. 0 in (*Z*)-*N*-hydroximine sulphate esters [8] (Figure 1). The structural diversity of glucosinolates is mainly due to the different substituents possible at the side-chain position R, which can be very variable [9]. More than 132 types of GSLs have been uncovered so far [10,11]. Table 1 lists all known GSL compounds identified to-date in *Eruca* species [7,12,13]. 

The GLs are naturally hydrolyzed by the myrosinase enzyme or enteric microflora giving rise to a number of products among which isothiocyanates are known for their antibacterial and antifungal properties applied to biofumigation [12,14,15], and also to efficiently reduce risks of degenerative diseases such as cancer [16]. It has been speculated that the isothiocyanates (ITCs) like sulforaphane (4-methylsulfinylbutyl), obtained from hydrolysis of glucoraphanin (4-(methylsulfinyl)butyl-GSL), are in great part responsible for the protective effects of cruciferous vegetables by the induction of Phase II enzymes. These enzymes have a chemoprotective effect, since they block chemical carcinogenesis, thus helping to prevent the onset of cancers [17,18,19].

The glucoraphanin, glucosativin (4-mercaptobutyl-GSL) and glucoerucin (4-(methylthio)butyl-GSL) have been identified as the most abundant GLs in leaf rocket [4]. Glucosativin and glucoerucin breakdown products are thought to contribute most to pungency and flavour in rocket [13]. The glucoerucin (GER) could undergo a high yielding transformation into GRA by chemoselective oxidation using hydrogen peroxide [20] and boosted our interest for GER and its ITC-derivative, erucin (ERN, 4-methylthiobutyl ITC). Previous investigation has shown that ERN induces apoptosis selectively in human leukemia cells, but not in nontransformed T lymphocytes, thereby being a promising new agent in cancer therapy [21].

It is possible, although speculative, that the ability of ERN to induce apoptosis selectively in cancer cells, unlike SFN, may be at least partially related to its unusual capability to act as a hydroperoxide-scavenging preventive antioxidant [22]. 

Further investigation is in progress to disclose possible relationships between the fine antioxidant/prooxidant balance of ERN and its selective pro-apoptotic behaviour. Other GSLs have also been identified within rocket tissue, for example diglucothiobeinin [23], 4-hydroxyglucobrassicin) [24] and 4-methoxyglucobrassicin [25]. 

In relation to the total phenolic acid content, rocket tissues also contain significant levels of polyglycosylated flavonoids, which are antioxidants poorly absorbed in the proximal gastrointestinal tract and are therefore likely to reach the colon in substantial quantities, protecting the colonic epithelium from free radical attack [26]. Previous works showed variability for the total phenolic compounds (4474.5 to 32,700 μg·g^−1^ dw) and demonstrated that rocket leaves are an excellent source of these compounds [4], being even richer than the commercial broccoli florets [27]. Taking into account the aforementioned it is therefore important to characterise the content of bioactive compounds in new or reintroduced cultivars. However, the quantification of the glucosinolate and/or phenolic acid content by the standard methods is expensive and time-consuming, and in addition, specialised personnel are needed. 

Numerous techniques have been employed for quantification of these compounds, such as thin-layer chromatography, palladium test, UV spectroscopy, reverse phase by high performance liquid chromatography (LC), thermospray LC with tandem MS in the two most common interfaces (ESI or APCI), capillary gas chromatography (GC-MS and GC-MS-MS), high speed counter current chromatography or supercritical fluid chromatography [28,29,30]. The high cost and labour input required for obtaining the glucosinolate content by LC or total phenolic acid content by UV–visible spectrometry are serious handicaps to analyse large sets of samples, which is usually necessary to identify the target genotypes in screening programmes. In contrast, the use of fast analytical techniques such as near-infrared spectroscopy (NIRS) results in many advantages, since analysis can be carried out with a considerable saving of time, at a low cost and without using hazardous chemicals. NIRS has been widely used for decades for qualitative and quantitative analysis in agriculture and food research, and many authors have used this technique for determining the glucosinolate content in seeds, [29,31,32,33], leaves [12,34,35] and flowers [36] from a wide range of cruciferous species. Therefore, it may be expected that this technique coupled with chemometric tools could provide an alternative method to undertake the analysis of glucosinolates in rocket leaves.

This study is part of an ongoing breeding programme focused on obtaining varieties of rocket with enhanced health properties for human nutrition [30]. The main objective was to test the potential of NIRS for predicting the total and individual major glucosinolate content, as well as total phenolic acid content found in the rocket leaves. In addition, we provide some knowledge about the mechanism used by NIRS for determining these compounds successfully in the leaves of this species.

## 2. Results and Discussion

### 2.1. Reference Values

Thirteen glucosinolates belonging to three chemical classes were detected in rocket leaves: seven aliphatic compounds (glucoerucin, gluraphanin, gluconapin, glucoiberverin, progoitrin, gluconapoleiferin, glucobrassicanapin and mercaptobutyl), one aromatic (gluconasturtiin) and four indole compounds (4-hydroxyglucobrassicin, 4-methoxyglucobrassicin, glucobrassicin and neoglucobrassicin). The GL profile found in these accessions was similar to the profiles reported by other authors in rocket leaves [4,25,37,38,39].

The ranges, means and standard deviations of the total and individual glucosinolates used in this study are summarised in Table 2. Individual plants exhibited TGSL concentrations that ranged from 4.86 to 44.65 μmol·g^−1^ dw, and a mean value of 15.6 μmol·g^−1^ dw. These concentrations are similar to those contents previously found in rocket with values varying from 11 to 28.24 μmol·g^−1^ dw but lower than those found in rocket sprouts which reached up to 55.4 μmol·g^−1^ dw [39].

The GRA aliphatic glucosinolate varied from 0.13 to 27.53 μmol·g^−1^ dw, with a mean content of 9.11 μmol·g^−1^ dw and representing 38.66% of the TGSL. These results agree with those reported by Kim and Ishii [25] and Villatoro-Pulido [4] which found values from 1.25 to 6.1 μmol·g^−1^ dw, but higher values have also been reported by Bennet et al [37,38] with 32 μmol·g^−1^ dw. 

The GRA aliphatic glucosinolate varied from 0.13 to 27.53 μmol·g^−1^ dw, with a mean content of 9.11 μmol·g^−1^ dw and representing 38.66% of the TGSL. These results agree with those reported by Kim and Ishii [25] and Villatoro-Pulido [4] which found values from 1.25 to 6.1 μmol g^−1^ dw, but higher values have also been reported by Bennet et al [37,38] with 32 μmol·g^−1^ dw. 

Glucosativin was the indole glucosinolate that showed the highest mean content of all, representing 41% of the TGSL, ranging from 0.25 to 18.61 μmol g^−1^ dw followed by glucoerucin and glucoraphanin. 

The GER aliphatic glucosinolate varied from 0.14 to 8.01 μmol·g^−1^ dw. These concentrations are similar to values previously reported in rocket which ranged from 0.14 to 4.03 μmol·g^−1^ dw [4,25].

Total phenol content ranged from 2.30 to 12.3 mg·GAE·g^−1^ dw. The values found in this study were lower than those previously reported in rocket leaves where the concentrations varied from 4.47 to 32.7 mg·GAE·g^−1^ dw, and in seeds and sprouts from commercial broccoli cultivars with values reaching 27.97 mg·GAE g^−1^ dw [39].

Table 3 shows the correlations among TGLs, individual GLs and TPC and their associated *p*-values. The TGLs were correlated significantly with GRA and GLSAT (R^2^ = 0.83 and R^2^ = 0.70 at *p* < 0.01, respectively), which were the most abundant glucosinolates found in *Eruca* accessions (Table 2 and Table 3).

A moderate negative correlation was found between GER and GRA (R^2^ = −0.53, *p* < 0.01), suggesting that the biosynthesis of both molecules is interrelated. In *E. sativa*, GRA is derived by a side-chain modification of GER [38]. The structure of both compounds is identical, except for the presence of an oxygen atom on the side-chain sulfur (Table 1). GRA also had a moderate positive correlation with GLSAT (R^2^ = 0.476, *p* < 0.01). A weak positive correlation was found between GER and TPC (R^2^ = 0.33, *p* < 0.05). 

The presence of a negative correlation between GRA and GER, and a positive correlation between GRA and GLSAT is also of potential importance. These results suggest that indirect selection for high GRA and GLSAT is possible, but in detriment of GER.

In disagreement with our work, a recent study performed on accessions of *Eruca sativa* [40] indicated that no significant correlation was found between GRA and GER. Furthermore, no significant correlations were observed for these GLs with any sensory attribute.

These results underline the importance of knowing the relationships between phytochemical compounds in order to be taken into account in further breeding programs to find the adequate profile according to the different market preferences.

### 2.2. Spectral Data Pre-Treatments and Equation Performances

#### 2.2.1. Second Derivative Spectra of Rocket Leaf

**Figure 2:**
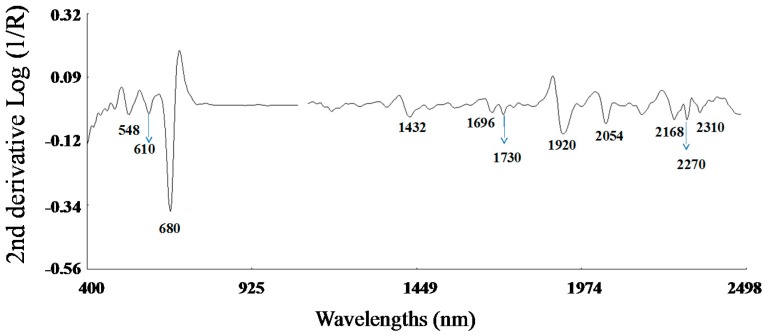
Second derivative spectra (2, 5, 5, 2; SNV + DT) of the raw optical data for rocket samples in the range from 400 to 2500 nm.

The application of the second derivative and standard normal variate and de-trending algorithms to the raw spectra (Log 1/R, Figure 2), resulted in substantial correction (Figure 2) of the baseline shift caused by differences in particle size and path length. Peaks and troughs in Figure 2 correspond to the points of maximum curvature in the raw spectrum, and it has a trough corresponding to each peak in the original. The increase in the complexity of the derivative spectra resulted in a clear separation between peaks which overlap in the raw spectra. 

The bands in the visible region at 548 and 670 nm are due to electronic transitions in the green and red, respectively. Thus, the band at 670 nm has been assigned to chlorophyll [41], which near 680 nm has a strong inverse correlation with sugar content [42]. In the NIR segment of the spectrum, the main absorption bands were displayed at 1920 nm, which has been attributed to O-H stretch plus O-H deformation; 2054 nm related to N-H stretch of amides; 2270 nm which has been assigned to O-H plus C-C stretch groups [43] of cellulose, and at 2310 nm related to C-H stretching and combination bands of the methylene groups [44]. Other minor absorptions were due to the first overtone of O-H stretching (1432 nm), S-H stretch first overtone or C-H stretch first overtone of CH_3_ groups (1696 nm), and C-H stretching by methylene groups (1730 nm).

#### 2.2.2. Calibration Equation

Table 2 shows the cross-validation statistics for the best-fitting equations obtained using FNS-6500.The coefficient of determination for cross-validation (R^2^CV) oscillated from 0.64 (GLSAT) to 0.94 (GER). For RPD values, the values obtained varied between 1.62 (GLSAT) to 3.99 (GER). 

The statistics of the external validation for the different quality compounds, including standard errors of performance (SEP) and R^2^VAL values for the equations of best fit obtained for each of the traits are shown in Table 4. The SEP values obtained in the validation were lower than their respective SD, indicating that NIRS is able to determine these traits in rocket leaves. The standard errors of the glucosinolate predictions reported in previous studies ranged from 0.05 to 18.74 μmol·g^−1^ dw for *Brassica* cultivars and Indian mustard seeds. The percentage of error for total and individual glucosinolates in this work is similar to the errors previously reported for the aforementioned matrices such as leaves, flowers and seeds [33,34,35,36,45].

According to Williams and Norris [46] depending on the R^2^ value from the external validations, models can be classified as models that can be used to discriminate between low and high sample values, in our study those obtained for TGLs, GSAT and GER; models that can be used for rough predictions of samples, calibrations obtained for GRA and models with good correlations, as was the case of the calibration obtained for TPC (Table 4). 

On the basis of guidelines for interpretation of RPD from external validation [46], if this ratio exceeds a value of 3 the calibration equation is very significant, this being obtained in this study for total phenolic compounds; the ratios between 1.5 *<* RPD_p_ *<* 2.5 characterize the equations as suitable for screening purposes, which was obtained for GER, TGLs GLSAT and GRA (Table 4). 

**Figure 3:**
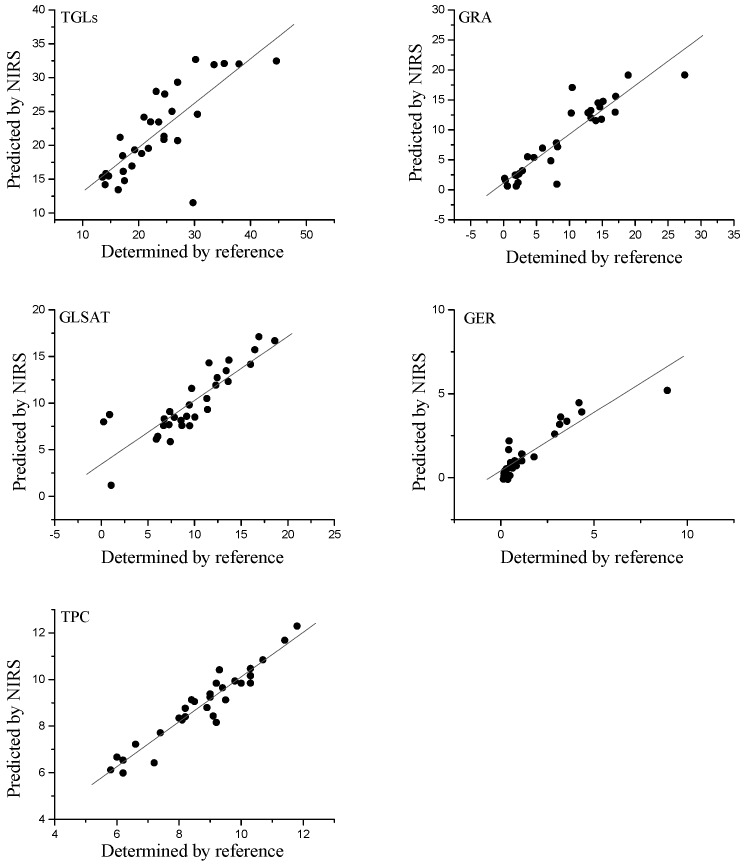
External validation scatter plot for near infrared predicted values versus reference values for glucosinolates and total phenolic acid content in rocket leaves.

Figure 3 shows the relationship between the predicted reflectance spectroscopy in the near infrared (NIRS) and reference values for the glucosinolates and total phenolic content in the validation set samples. 

In terms of RER coefficients, the predictive ability of the equations in this work extended from 6.48 to 14.89 (Table 4). For GRA, GER and TPC, the validation yielded RER (9.95–14.95) values, which indicated models with good precision. For TGLs and GLSAT the external validation yielded RER (6.48–7.78) values, indicative of models that could be used for screening purposes, which could be very useful as a selection tool in rocket breeding programmes and quality control [47]. 

Previous studies reporting NIRS calibrations of glucosinolates in *Brassica* species have been performed mainly on rapeseed seed, because of the commercial interest of this species. Thus, Biston [31] reported R^2^ VAL values of 0.99 for total glucosinolates, independently of the different reference methods used (palladium, glucose, gas-liquid chromatography or LC). Daun et al. [32] also reported predictions for total glucosinolates with coefficients of determination that varied from 0.74 to 0.82, and ratios of the standard error of prediction (SEP) performed on external validation to the SD of the reference values (RPD), that ranged from 1.36 to 2.29. Other authors [29] developed multi-product calibrations for individual and total glucosinolates considering simultaneously different *Brassica* species. These authors demonstrated the validity of the technique in approaches like that, reporting high R^2^CV values for individual (gluconapin = 0.89; sinigrin = 0.90; progoitrin = 0.86) and total glucosinolates (0.99) predictions. Mika [48] in a work performed on *Brassica napus*, reported R^2^ VAL value for total glucosinolates on external validation (0.84) and RER value (7.5) slightly higher than obtained in this work. In a previous work performed on *Brassica juncea* (L. Czern. & Coss.) seed [33], they obtained R^2^ VAL values that ranged from 0.82 to 0.95 for total and individual glucosinolates. It has to be noted that all the above referenced works, showed prediction accuracies that were similar or slightly higher than those reported for leaves in this work, in spite of the lower concentrations of these compounds in rocket leaves. 

The determination coefficients of cross-validation obtained in this study for GER (0.93) and GRA (0.82) were higher than those reported on *Brassica napus* [34] and *Brassica oleracea* leaves [35] (gluconapin: 0.73; glucobrassicin: 0.81; progoitrin: 0.78; glucoalyssin: 0.37; glucobrassicin: 0.41; gluconapin: 0.70; gluconasturtiin: 0.62; neoglucobrassicin: 0.60), similar to reported for GLSAT (0.65), and lower than those reported for total glucosinolates (TGLs > 0.83) in our work (TGLs: 0.70). 

The potential of vis-NIRS for determining these compounds in freeze-dried broccoli has also been examined [36]. The R^2^CV reported in the aforementioned study for different GLs (glucobrassicin: 0.89; methoxyglucobrassicin: 0.69; neoglucobrassicin: 0.68; total glucosinolates: 0.73) were similar to those obtained in our work, but lower than those reported for glucoraphanin (GRA: 0.40). This fact is remarkable for the importance to select accessions with high GRA contents for the anticarcinogenic properties of sulforaphane (an isothiocyanate obtained from hydrolysis of glucoraphanin). 

Previous calibration models have also been developed for non-destructive estimation of total phenol content in intact rapeseed mustard seed by Fourier transform near infrared spectroscopy [49]. The optimal models for total phenol were achieved with coefficient of determination R^2^CV of 0.96 and RPD_CV_ values of 4.98, these coefficients were higher than those obtained in the present work. The lower total phenolic concentration in rocket leaf (2.30–12.30 mg·GAE·g^−1^ dry weight) in comparison with those detected in rapeseed-mustard seeds (7.8–23.9 mg·GAE·g^−1^ fresh weight) could be on the basis of the lower accuracy of the calibration model for these compounds.

#### 2.2.3. Modified Partial Least Square Loadings

**Figure 4:**
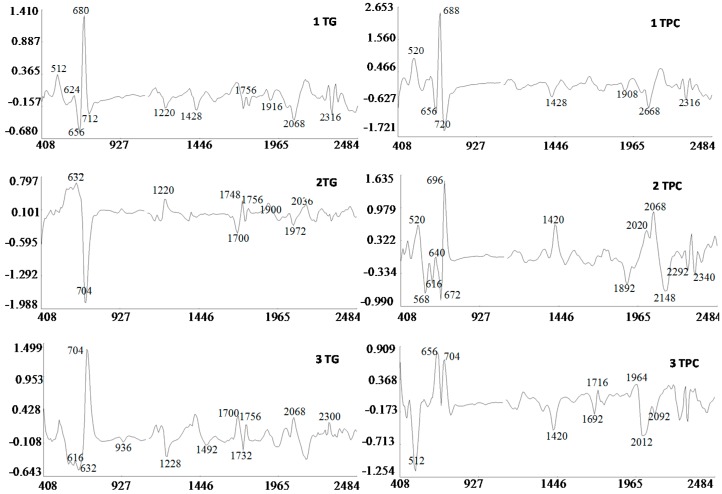
MPLS loading plots for factor 1, 2 and 3 of the 2, 5, 5, 2 (SNV + DT) transformation for total glucosinolate and phenolic acid content using near infrared reflectance spectroscopy.

Figure 4 shows the three MPLS loading plots of the TGL and TPC equations. The first PLS term of the equations was strongly influenced by absorption bands characteristic of pigments localized between 400 and 700 nm. Thus, carotenoids found in the genus *Eruca* might be influencing the absorption band at λ_max_ = 470 nm including β-carotene, and xanthophylls like lutein [4]. High absorbance observed at 670 nm is indicative of red absorbing pigments, particularly chlorophyll that gives the fruit its characteristic green color [50]. The NIR region of the spectrum showed characteristic absorption bands at 1212 nm which is in the spectral segment assigned to C-H stretch second overtone and water and also by absorption bands at 1724 nm and 1764 nm corresponding to C-H stretch first overtone and C-H combinations at 2308 nm and 2348 nm. Those wavelengths corresponding to absorptions by plant pigments (624 nm), C-H stretching (1740 nm), N-H stretching by amides (2052 nm) and O-H stretch/OH deformation hydroxyl (1916 nm) [35] highly influenced the second factor of the equation (Figure 4). The third factor was mainly modelled with those wavelengths corresponding to pigments (720 nm), N-H stretching of amides (2084 nm), C-H combinations of CH_2_ groups (2324 nm), O-H stretch/OH deformation hydroxyl (1924 nm) and C-H stretching (1756 nm and 1212 nm). 

Because the R group of glucosinolates (Figure 1) is derived from amino acids, it is possible that some correlation between the protein and the total glucosinolate content of the leaf exists (peak at 2084 nm, Figure 4). In the case of phenolic content, these compounds possess one or more aromatic rings with one or more hydroxyl groups, mainly related with combination bands of the –OH functional group, C–H aromatic second overtones and C–H third overtones (regions from 1415 to 1512 nm and from 1512 to 2035 nm) [43,51].

## 3. Materials and Methods

### 3.1. Plant Material and Greenhouse Experiments

Fifty-two accessions of *Eruca* were acquired from different European genebank collections and collected from different countries of the world. The vegetal material consisted of: one accession of *Eruca stenocarpa*, one accession of *Eruca vesicaria* subsp. *longirostris*, 10 accessions of *Eruca vesicaria* subsp. *vesicaria* and 40 accessions of *Eruca vesicaria* subsp. *sativa*. 

These accessions are part of a germplasm collection located at the IFAPA-La Mojonera, Almería (Southern Spain). Seeds were germinated in Petri dishes for 48 h at 25 °C. Pots were placed in a greenhouse under natural light at 27/18 °C (day/night) and a relative humidity of 50/70% (day/night). When the plants reached 8–12 cm, they were transferred to a field in Córdoba, Spain (37°51″42′ N, 04°48″00′ W; 220 m a.s.l.). The experiment was designed as a randomized complete block consisting of rows of 5 meters in length with three replicates each.

### 3.2. Sample Pre-Treatment and Storage

Leaves from 10 randomly selected plants per replicate (52 accessions × 3 replicates) were harvested eight weeks after transplanting and on the same day. They were washed, weighed to assess their biomass, and placed in Ziploc-type freezer bags at −20 °C for post-harvest storage. The samples were freeze-dried and ground using a pestle and mortar.

### 3.3 GLs Analysis by Liquid Chromatography with Ultraviolet Absorbance Detection (LC-UV)

A hundred mg of freeze-dried sample was heated at 75 °C for 15 min in 2.5 mL of 70:30 methanol–water and 200 μL of 10 mM sinigrin as an external standard (sinigrin hydrate, 85440 Fluka, St. Louis, MO, USA) according to the ISO norm (ISO 9167-1, 1992). A second extraction was applied after centrifugation (5 min, 5 × 10^3^*g*) using 2 mL of 70:30 methanol-water. The combined GLs extracts were pipetted (1 mL) onto the top of an ion-exchange column containing 1 mL of Sephadex DEAE-A25 (40-125 μm bead size, 30000 Da exclusion limit). Desulfation was carried out by addition of 75 μL of purified sulfatase (EC 3.1.6.1, type H-1 from *Helix pomatia*) (Sigma-Aldrich, St. Louis, MO, USA) solution. Desulfated GLs were eluted with 2.5 mL of Milli-Q (Millipore, Bedford, MA, USA) ultrapure water and analyzed with a 600 HPLC instrument (Waters) equipped with a model 486 UV tunable absorbance detector (Waters, Milford, MA, USA) fixed at a wavelength of 229 nm. Separation was carried out using a Lichrospher 100 RP-18 in Lichrocart column (125 mm × 4 mm i.d., 5 μm particle size, Merck, Darmstadt, Germany). The HPLC chromatogram was compared to the desulfo-GL profile provided by three certified reference materials recommended by the U.E. and ISO (CRMs 366, 190 and 367) (Commission of the European Communities, report EUR 13339 EN, 1-75) [52].

### 3.4. Determination of the Total Phenolic Fraction

The concentration of total phenolic compounds (TPC) was estimated by a modified version of the Folin–Ciocalteu method [53], using gallic acid as standard, for which a calibration curve was run with solutions of 50, 100, 200, 300, 400, 500 and 600 mg/L of this compound. A 0.06 mL aliquot of extract 1.58 mL of distilled water, 0.1 mL of Folin-Ciocalteu reagent and 0.3 mL of Na_2_CO_3_ (20% *w*/*v*) were mixed and heated at 50 °C for 5 min. After 30 min, the absorbance was measured at 765 nm against a blank similarly prepared, but containing 70:30 ethanol–water mixture (pH 3.2) instead of extract. Sodium carbonate (Panreac, Spain), Folin–Ciocalteu reagent (FCR) and gallic acid (both from Sigma-Aldrich) were used to determine the total phenol fraction. The absorbance was measured with a ThermoSpectronic UV-visible Spectrometer (Thermo Fisher Scientific, Waltham, MA, USA).

### 3.5. NIRS Analysis Calibration and Validation Development 

The freeze-dried rocket samples were scanned using the FNS-6500 scanning instrument (FOSS NIRSystems, Silver Spring, MD, USA) in a small ring cup (3.75 cm φ) using the spinning sample module. Spectra were collected on all samples in the reflectance mode, acquiring their spectra over a wavelength range from 400 to 2500 nm (visible and near infrared regions). Samples were scanned in duplicate and the average spectrum was used to develop the multivariate models. Reflectance data were stored as log (1/R) (R = reflectance) at 2 nm intervals (1050 data points). 

Before developing NIRS calibrations, the structure and spectral variability of the sample population was determined using the CENTER algorithm included in the WinISI software. This programme performs an initial principal component analysis (PCA) to calculate the centre of the population and the distance of samples (spectra) from that centre in an n-dimensional space using the Mahalanobis distance (GH); samples with a statistical value greater than three were considered outliers or anomalous spectra [54]. Thus, having ordered the sample set by spectral distance (from smallest to greatest distance to the centre), the 30 samples forming the validation set were selected by taking one of every 5 samples in the final 156 sample set; the calibration set thus comprised the remaining 126 samples. 

Calibration equations for physico-chemical were developed using the programme GLOBAL v. 1.50 (WINISI II, Infrasoft International, LLC, Port Matilda, PA, USA). Calibration equations were computed using raw optical data (log 1/R, where R is reflectance), or first or second derivatives of the log 1/*R* data, with several combinations of derivative (gap) sizes and smoothing [i.e., (0, 0, 1, 1; derivative order and segment , first smooth, second smooth); (1, 4, 4, 1); (1, 10, 10, 1); (2, 5, 5, 2); (2, 20, 20, 2)]. The regression method employed to correlate spectral information and quality compounds in the samples was modified partial least squares (MPLS). This regression method is a soft-modelling method [41,42] for constructing predictive models when the factors are many and highly collinear and allows a model to be calculated that was tested on external samples observing its prediction ability. The final objective of the mathematical procedure is to reduce the high number of spectral data points (absorbance values from 400 to 2500 nm every 2 nm, i.e. 1050 data) and to eliminate the correlation of absorbance values presented by neighbouring wavelengths [55]. Standard normal variate and detrend transformations (SNV-DT) were used to correct baseline offset due to scattering effects (differences in particle size among samples) [56]. Cross-validation was performed on the calibration set for determining the best number of terms to use in the equation, as well as to determine the ability of each equation to make predictions on unknown samples [57]. 

An external validation procedure in 30 independent samples was carried out to determine the accuracy and precision of the equations obtained in the calibration for each quality component. The coefficient of determination (R^2^) and standard error (SE) were calculated for both cross-validation and external validation. The predictive ability of the mathematical models was assessed in the external validation from the R^2^, the RPD, which is the ratio of the standard deviation for the validation samples to the standard error of prediction (performance) (SEP), and the RER, which is the ratio of the range in the reference data (validation set) to the SEP. 

Depending on the R^2^ value from the external validation, NIR models can be classified [37] as: models with a low correlation (0.26 < R^2^VAL < 0.49); models that can be used to discriminate between low and high values of the samples (0.50 < R^2^VAL < 0.64); models that can be used for rough predictions of samples (0.65 < R^2^VAL < 0.81); models with good correlations (0.82 < R^2^VAL < 0.90); and models with excellent precision (R^2^VAL > 0.90). 

The guideline used for setting performance calibrations stated that an RPD value of more than 3 is desirable for excellent calibration equations, whereas equations with an RPD of less than 1.5 are unusable [46]. In relation to the range error ratio (RER), it should ideally be at least 10 [47]. 

The mathematical expressions of these statistics are as follows:

$$\mathrm{RPD}=SD{\langle {\left[\left(\sum_{i=1}^{n}{\left(y_{i}-{\hat{y}}_{i}\right)}^{2}\right){\left(N-K-1\right)}^{-1}\right]}^{1/2}\rangle }^{-1}$$

where $y_{i}$ = lab reference value for the *i*^th^ sample; $\hat{y}$ = NIR measured value; *N* = number of samples, *K* = number of wavelengths used in an equation; *SD* = standard deviation.

$$\mathrm{RER}=range{\langle {\left[\left(\sum_{i=1}^{n}{\left(y_{i}-{\hat{y}}_{i}\right)}^{2}\right){\left(N-K-1\right)}^{-1}\right]}^{1/2}\rangle }^{-1}$$

where $y_{i}$ = lab reference value for the *i*^th^ sample; $\hat{y}$ = NIR measured value; N = number of samples, K = number of wavelengths used in an equation.

### 3.6. Statistical Analysis

Correlation analysis was assessed by the Pearson test among TGLs, GRA, GLSAT and TPC to determine significant relationships. Statistical analyses were performed using SPSS 13.0 (SPSS Inc., Chicago, IL, USA).

## 4. Conclusions

Results reported in this work show that NIRS is able to predict the glucosinolate and phenolic contents in the leaf rocket, with sufficient accuracy for screening purposes. Each sample that we analyzed by using the NIRS method took us approximately 1.5 min, and prediction results for the individual and total glucosinolate and phenolic contents were monitored instantaneously. The equations shown in this work have been recently applied to the evaluation of both compounds in 3000 individual plants of rocket. This has allowed the rapid identification and selection of the genotypes of interest, which would not have been possible by using the reference method. NIRS is thus ideal for mass screening programmes in large-scale plant monitoring and quality control.

## Figures and Tables

**Table 1:** Identified glucosinolates in *Eruca* species differing only in the side chain R.

R (Variable Side Chain) Group Structure	R-Group	Trivial Name
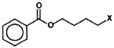	2-(Benzoyloxy) ethyl	-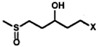	3-Hydroxy-5-(methyl-sulfinyl)pentyl	-
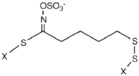	4-(β-D-glucopyranosyldisulfanyl) butyl	Diglucothiobeinin
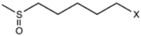	5-(Methylsulfinyl)pentyl	Glucoalyssin
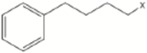	4-Phenylbutyl	Glucoamoracin
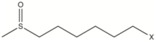	7-(Methylsulfinyl)heptyl	Glucoibarin
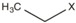	Ethyl	Glucolepiidin
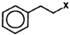	2-Phenylethyl	Gluconasturtiin
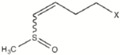	4-(Methylsulfinyl)-3-butenyl	Glucoraphenin
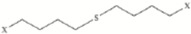	Dimeric 4-mercaptobutyl	DMB
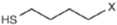	4-Mercaptobutyl	Glucosativin
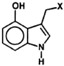	4-Hydroxy-3-indolymethyl	4-Hydroxyglucobrassicin
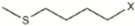	4-(Methylthio)butyl	Glucoerucin
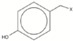	4-Hydroxybenzyl	Glucosinalbin
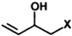	(*R*,*S*)-2-Hydroxy-3-butenyl	Progoitrin
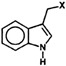	3-Indolymethyl	Glucobrassicin
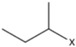	1-Methylpropyl	Glucocochlearin
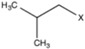	2-Methylbutyl	Glucojiabutin
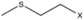	3-(Methylthio)propyl	Glucoiberverin
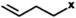	3-Butenyl	Gluconapin
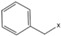	Benzyl	Glucotropaeolin
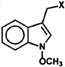	1-Methoxyindol-3-ylmethyl	Neoglucobrassicin
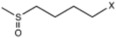	4-(Methylsulfinyl) butyl	Glucoraphanin
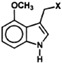	4-Methoxyindol-3-ylmethyl	4-Methoxyglucobrassicin

**Table 2:** Calibration and cross-validation statistics of glucosinolates (μmol·g^−1^ dw) and total phenolic content (mg·GAE·g^−1^ dw) for rocket leaves measured by FNS-6500. GRA: Glucoraphanin; GLSAT: Glucosativin; GER: Glucoerucin; TPC: Total phenolic content

Parameter	Range	Mean	SD ^1^	R ^2^C ^2^	SEC ^3^	R ^2^CV ^4^	SECV ^5^	RPDcv ^6^
**Total GLs**	4.86–44.65	23.56	7.32	0.79	3.34	0.70	4.02	1.83
**GRA**	0.13–27.85	9.11	6.41	0.94	1.52	0.82	2.72	2.37
**GLSAT**	0.25–18.61	9.66	3.92	0.86	1.44	0.64	2.42	1.62
**GER**	0.14–8.01	1.45	1.6	0.97	0.26	0.93	0.41	3.99
**TPC**	2.30–12.30	8.73	1.99	0.88	0.69	0.85	0.79	2.55

^1^ SD: standard deviation; ^2^ R^2^C: coefficient of determination in calibration. ^3^ SEC: standard error in calibration. ^4^ R^2^CV: coefficient of determination in cross-validation. ^5^ SECV: standard error of cross-validation. ^6^ RPDcv: ratio of the standard deviation to standard error of cross-validation.

**Table 3:** Pearson correlation coefficient (R^2^) among TGLs, individual GL and TPC in *Eruca* accessions.

Parameter	TGLs	GRA	GLSAT	GER	TPC
TGLs	1	0.83 **	0.70 **	−0.37 **	−0.04 n.s.
GRA		1	0.48 **	−0.53 **	−0.09 n.s.
GLSAT			1	−0.14 n.s.	0.09 n.s.
GER				1	0.33 *
TPC					1

TGLs: Total glucosinolates; GRA: glucoraphanin; GLSAT: glucosativin; GER: glucoerucin; TPC: Total phenolic content. n.s.: not significant; *: *p* < 0.05; **: *p* < 0.01.

**Table 4:** Reference values and external validation statistics of the NIRS calibrations for glucosinolate (μmol·g^−1^ dw) and total phenolic content (mg·GAE·g^−1^ dw) in rocket leaves.

Parameter	Reference Values (*n* = 30)			External Validation				
	Range	Mean	SD ^1^	R^2^VAL ^2^	SEP(C) ^3^	RPDp ^4^	RER ^5^	
Total GLs	13.52–39.88	23.56	7.63	0.61	4.80	1.59	6.48	
GRA	0.15–27.53	9.11	6.82	0.79	2.75	2.48	9.95	
GLSAT	0.35–17.52	9.66	4.53	0.60	2.36	1.92	7.78	
GER	0.20–7.34	3.20	1.90	0.59	1.22	1.56	14.89	
TPC	5.80–11.80	8.73	1.57	0.84	0.48	3.27	12.5	

^1^ SD: standard deviation; ^2^ R^2^VAL: coefficient of determination in external validation; ^3^ SEP(C): standard error of prediction corrected for bias; ^4^ RPDp: ratio of the standard deviation to standard error of prediction (performance); ^5^ RER: ratio of the range to standard error of prediction (performance).
